# Supplementary figures and images for: Long-range DNA end resection supports homologous recombination by checkpoint activation rather than extensive homology generation
Source: eLife. 2023 Jun 30;12:e84322. doi: 10.7554/eLife.84322 (PMC10400078; doi:10.7554/eLife.84322)

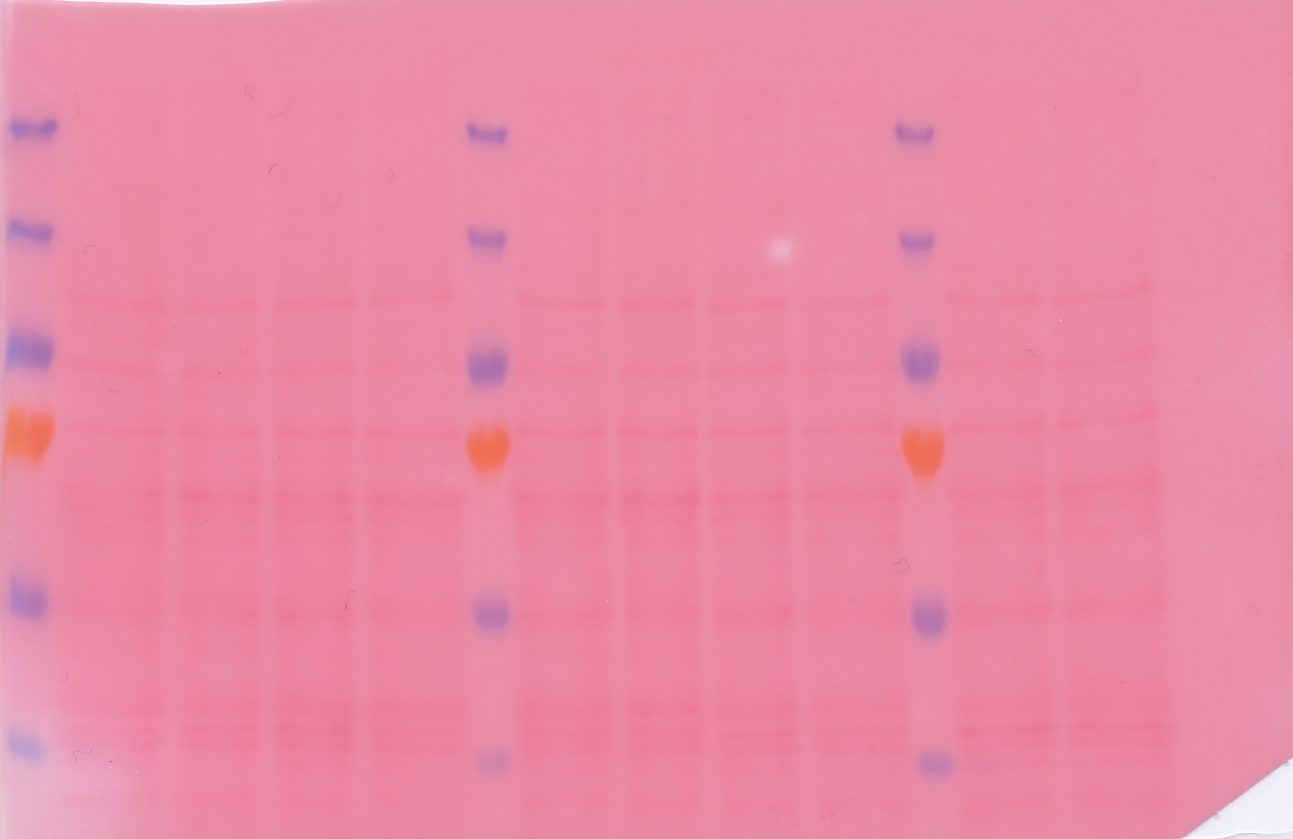

Supplement: Figure 2—source data 1. [file elife-84322-fig2-data1.zip › Figure 2- supplement 2/Fig2_S2- source data 2.jpg]

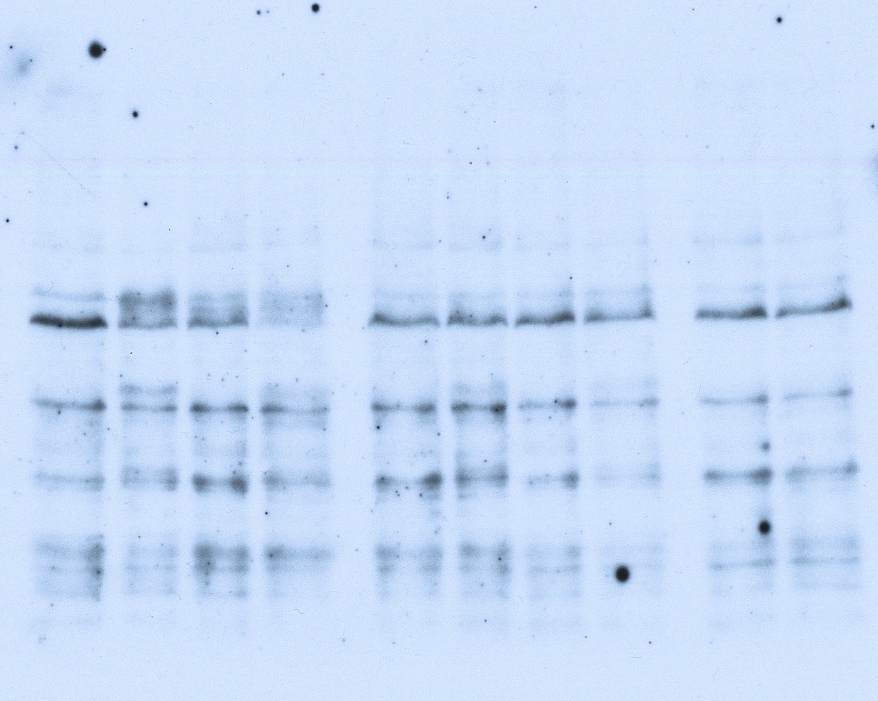

Supplement: Figure 2—source data 1. [file elife-84322-fig2-data1.zip › Figure 2- supplement 2/Fig2_S2- source data 1.jpg]

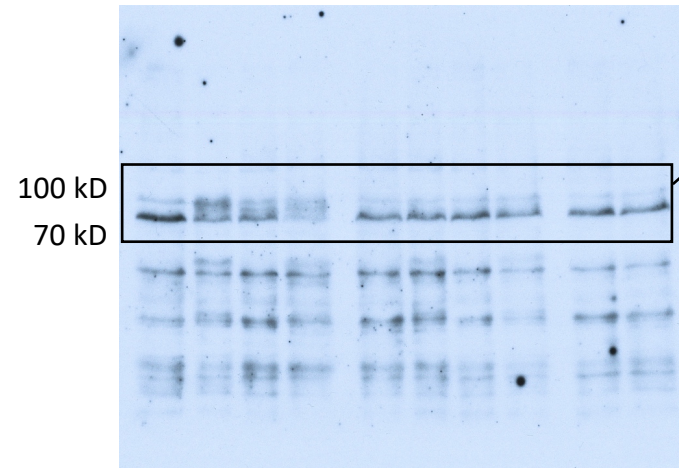

Regions cropped  
for Fig2\_S2

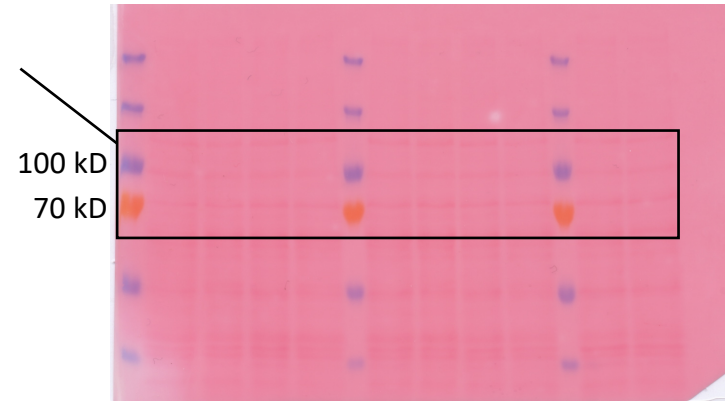

Supplement: Figure 2—source data 1. [file elife-84322-fig2-data1.zip › Figure 2- supplement 2/Fig2_S2- source data.pdf]

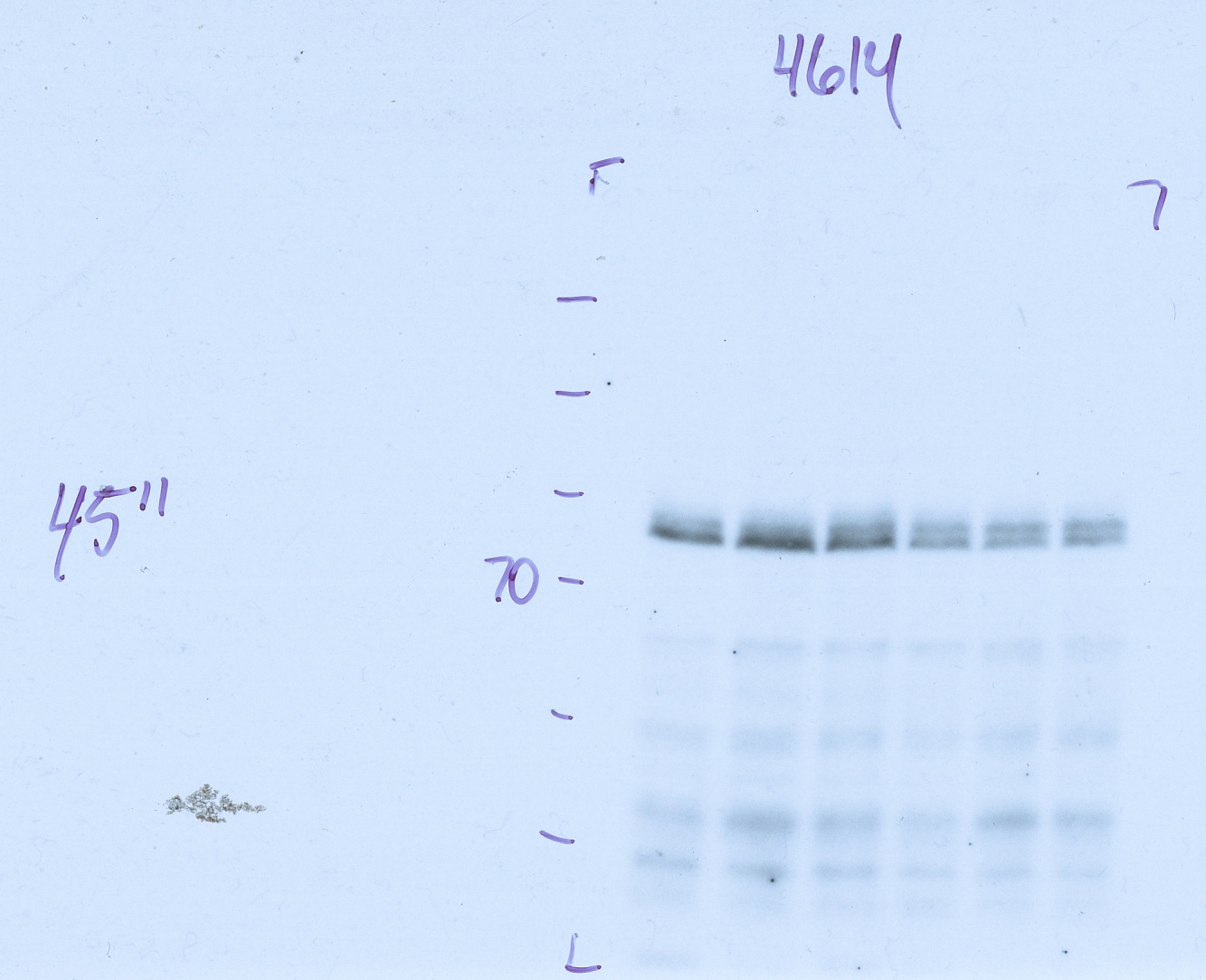

Supplement: Figure 2—source data 1. [file elife-84322-fig2-data1.zip › Figure 2D/Figure 2- source data (Inter exo1 sgs1 blot).jpeg]

WT

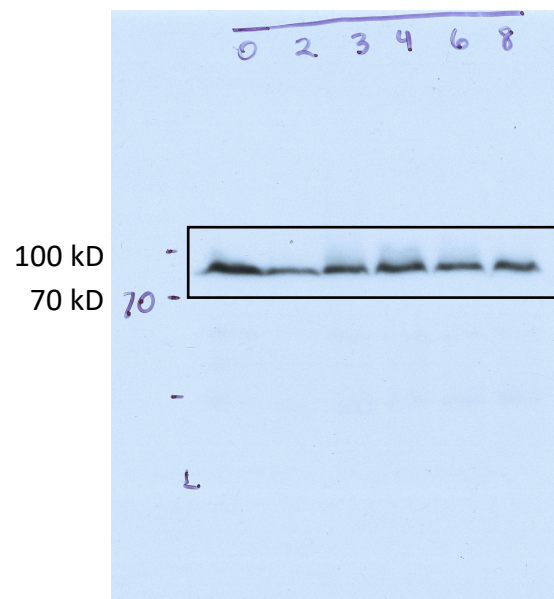

Regions cropped  
for Figure 2D

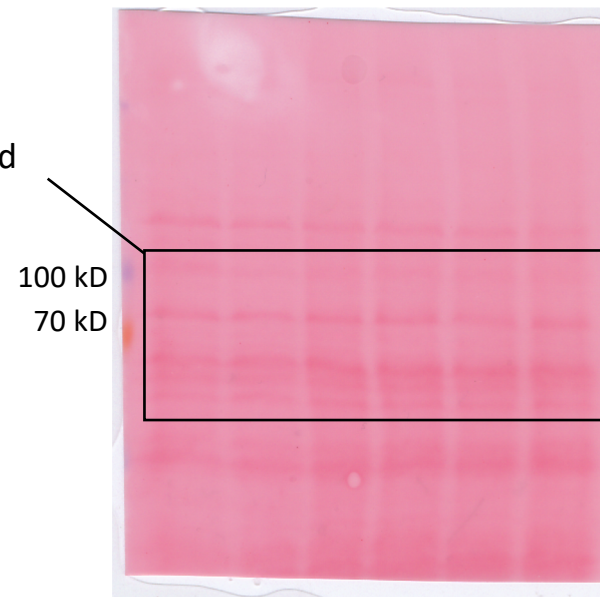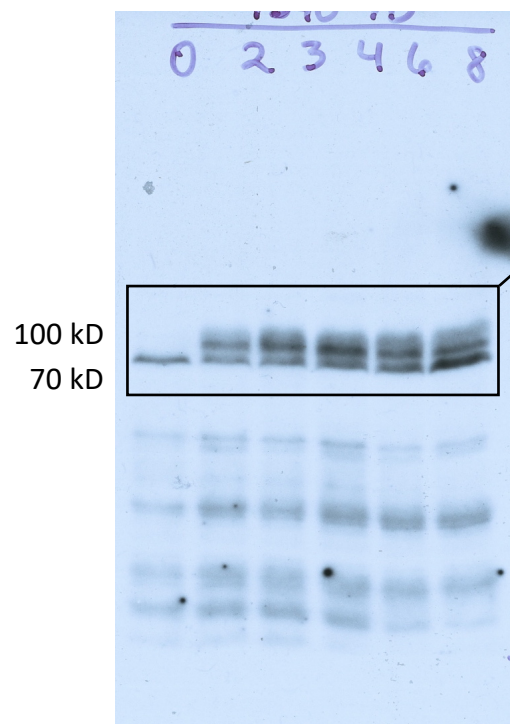

Regions cropped  
for Figure 2D

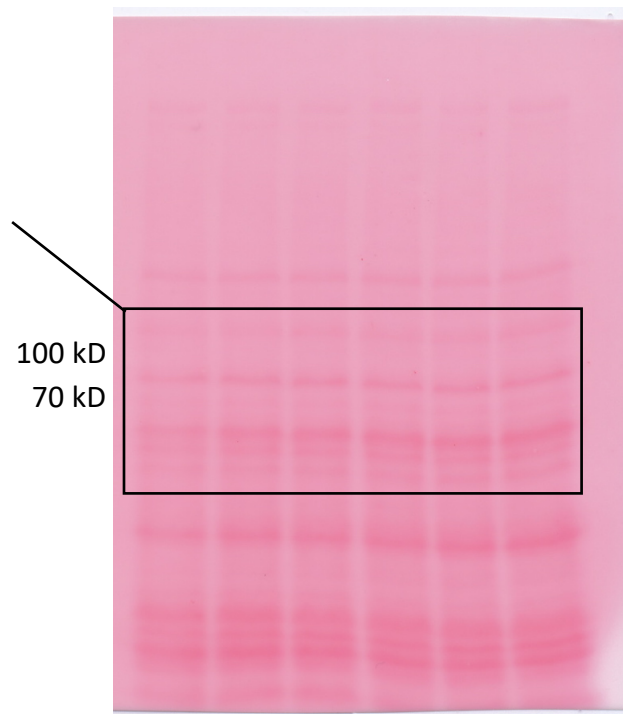

*exo1Δ sgs1Δ*

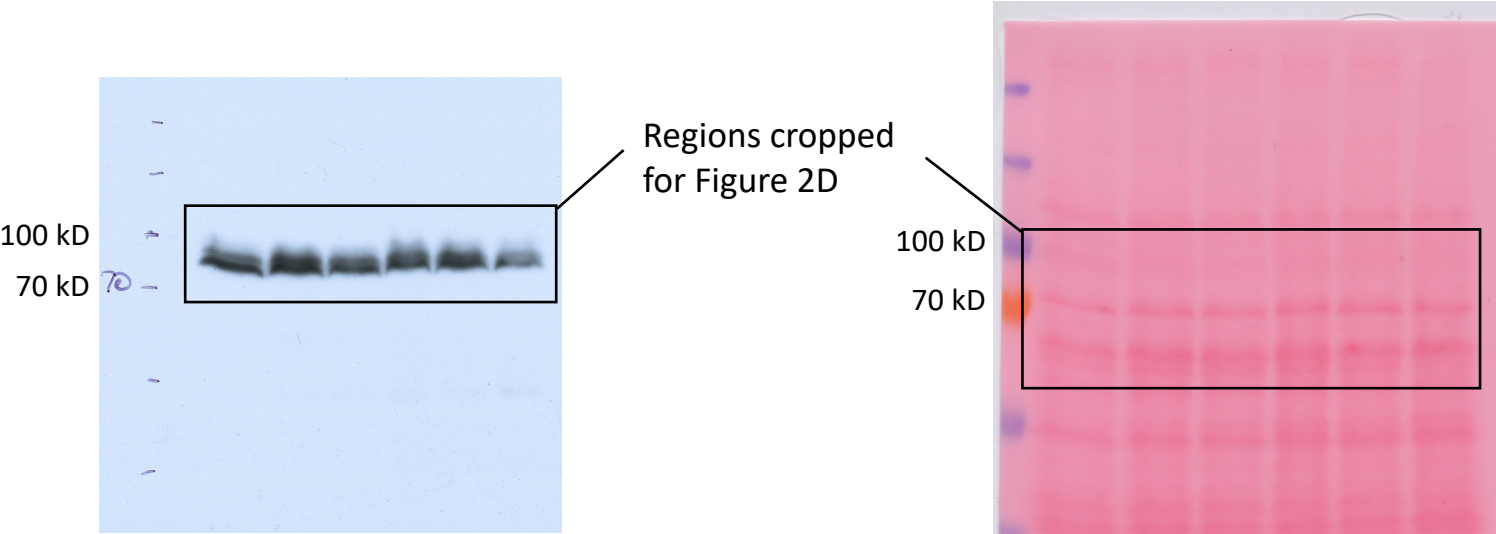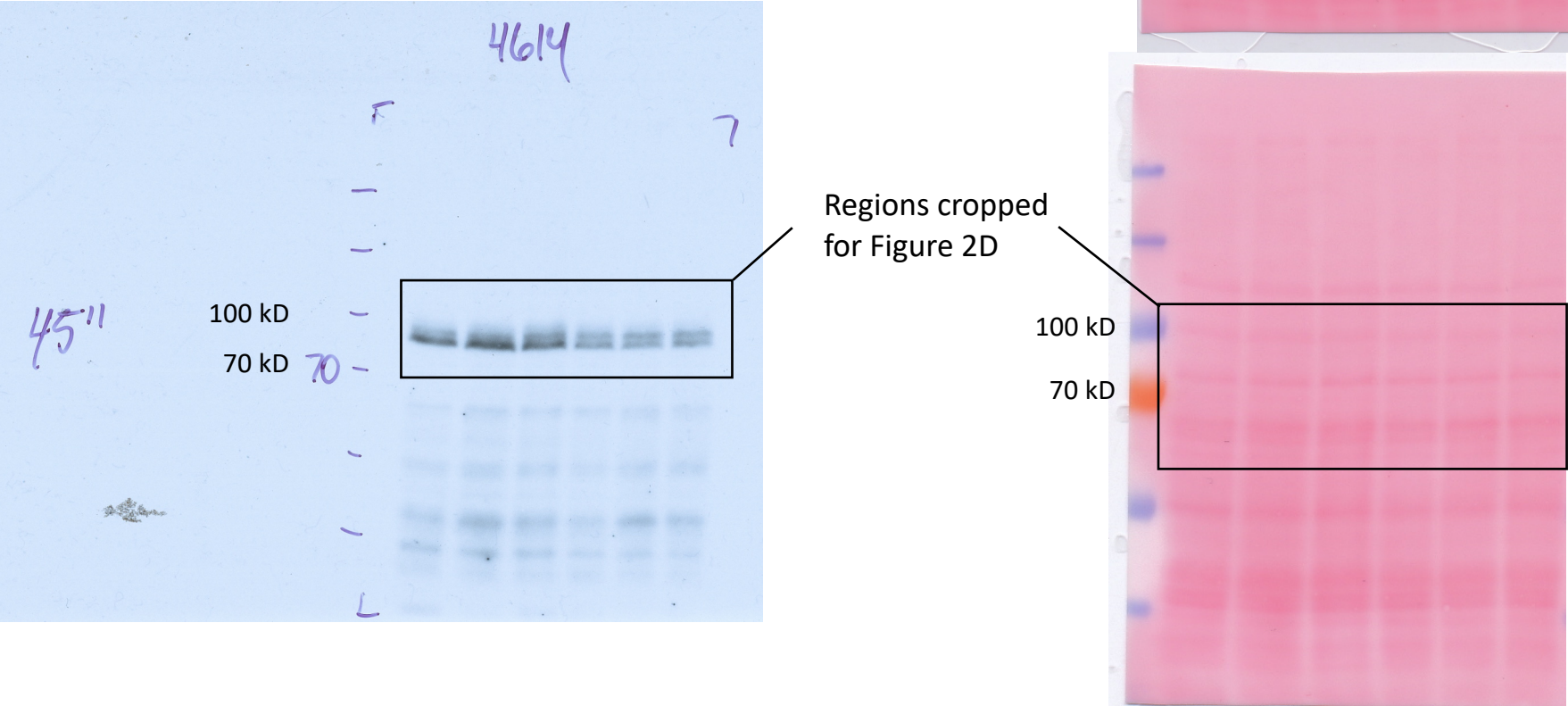

Supplement: Figure 2—source data 1. [file elife-84322-fig2-data1.zip › Figure 2D/Figure 2D- source data.pdf]

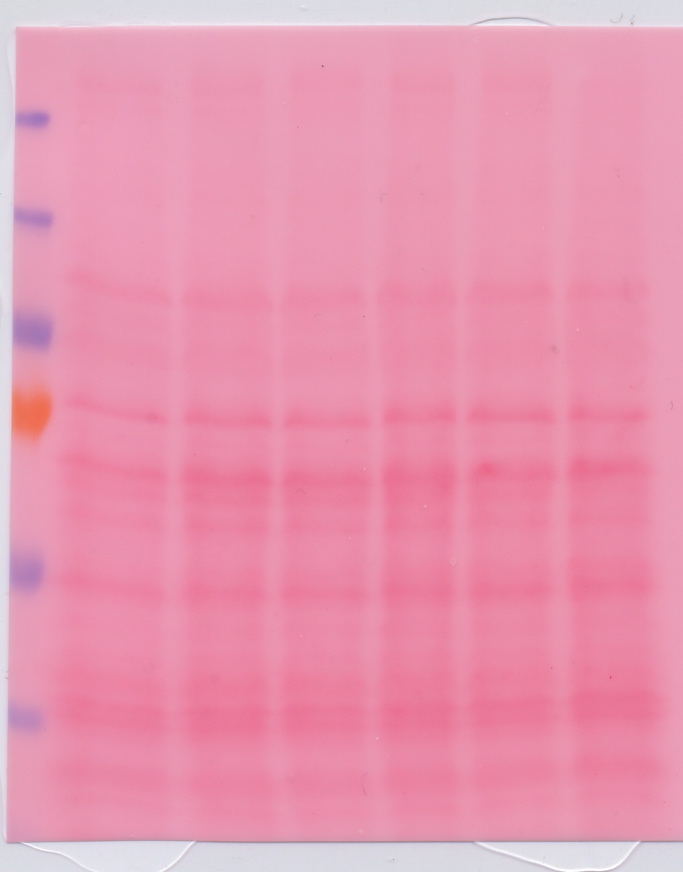

Supplement: Figure 2—source data 1. [file elife-84322-fig2-data1.zip › Figure 2D/Figure 2- source data (Intra exo1 sgs1 Ponceau stain).jpeg]

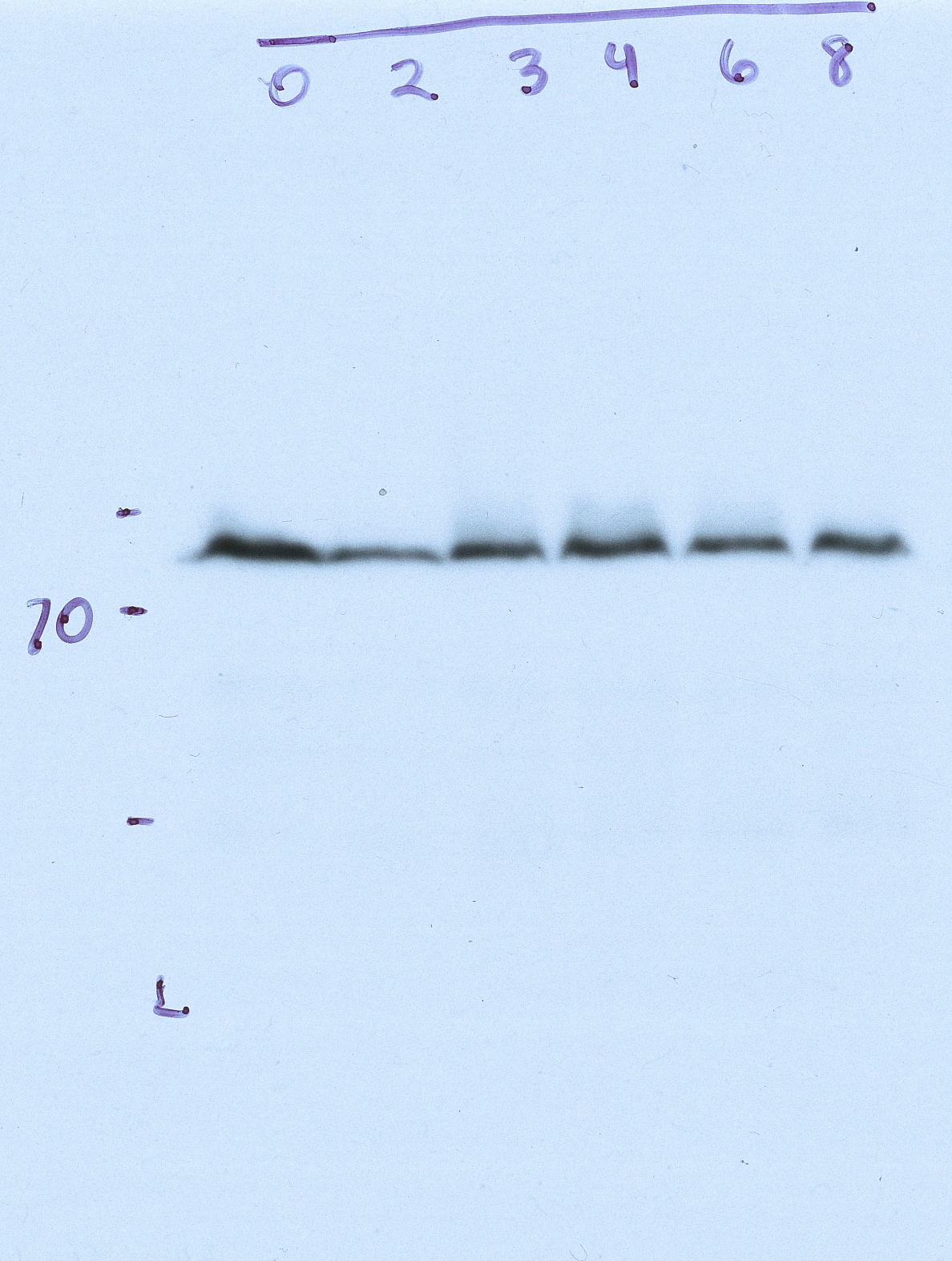

Supplement: Figure 2—source data 1. [file elife-84322-fig2-data1.zip › Figure 2D/Figure 2- source data (Intra WT blot).jpeg]

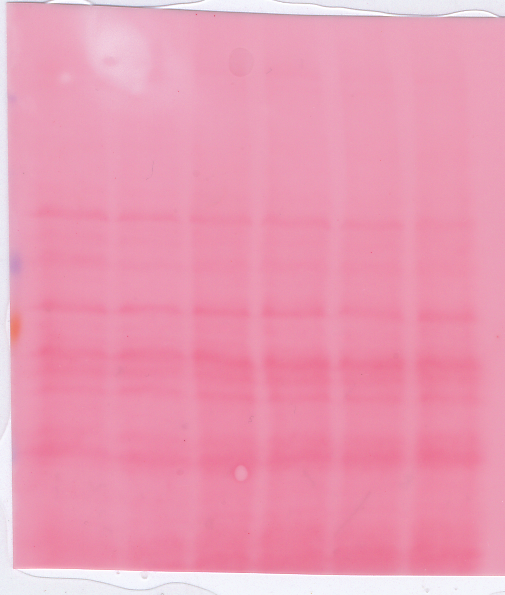

Supplement: Figure 2—source data 1. [file elife-84322-fig2-data1.zip › Figure 2D/Figure 2- source data (Intra WT Ponceau stain).tiff]

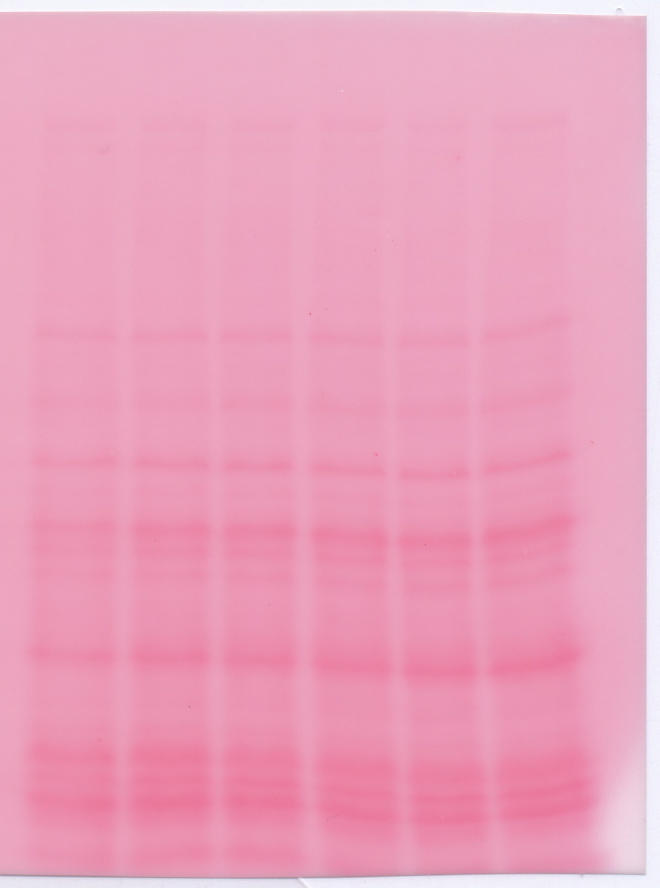

Supplement: Figure 2—source data 1. [file elife-84322-fig2-data1.zip › Figure 2D/Figure 2- source data (Inter WT Ponceau stain).jpeg]

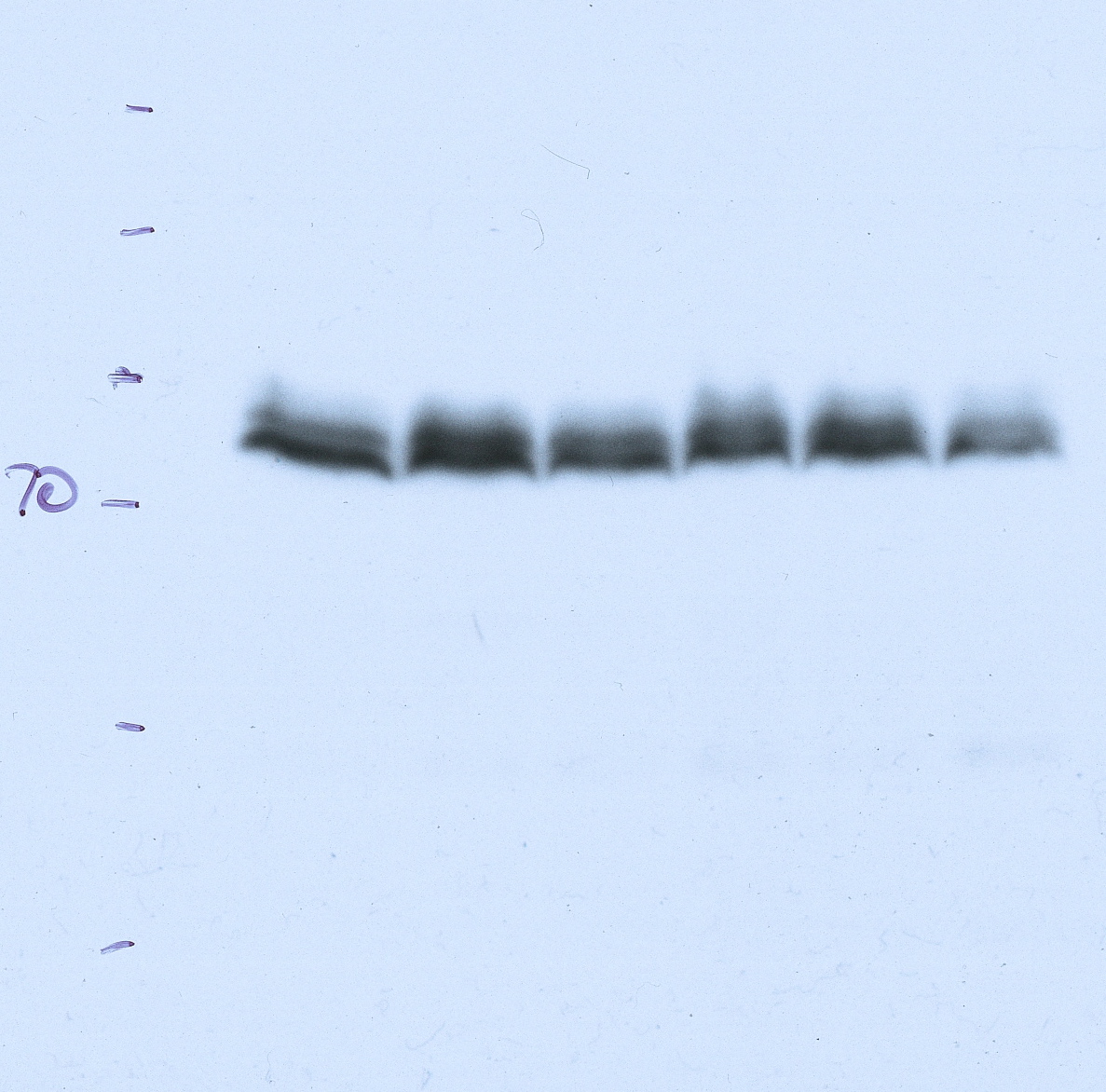

Supplement: Figure 2—source data 1. [file elife-84322-fig2-data1.zip › Figure 2D/Figure 2- source data (Intra exo1 sgs1 blot).jpeg]

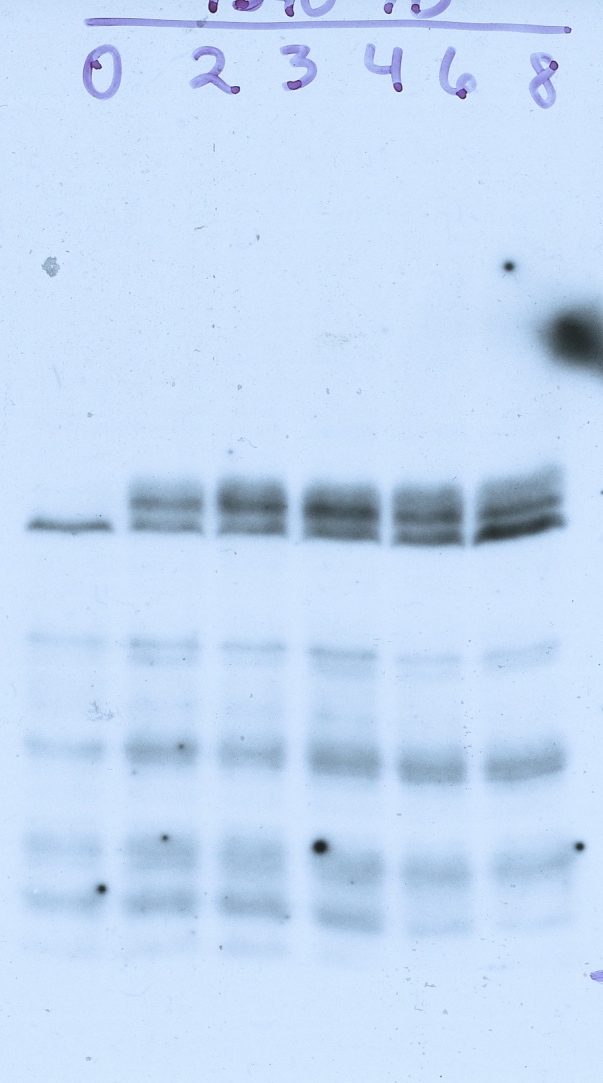

Supplement: Figure 2—source data 1. [file elife-84322-fig2-data1.zip › Figure 2D/Figure 2- source data (Inter WT blot).jpeg]

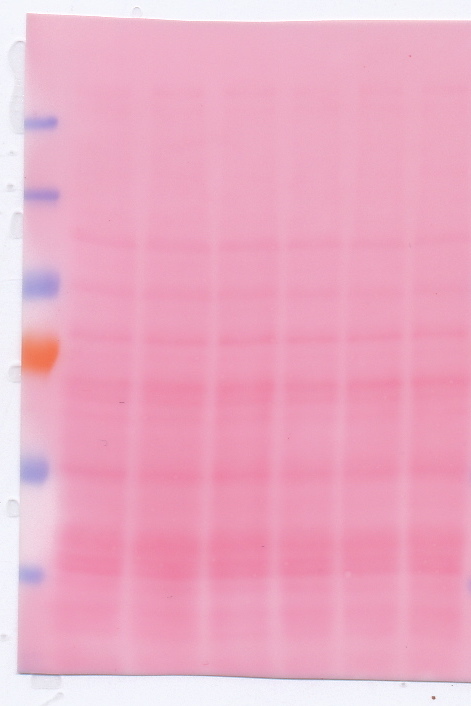

Supplement: Figure 2—source data 1. [file elife-84322-fig2-data1.zip › Figure 2D/Figure 2- source data (Inter exo1 sgs1 Ponceau stain).jpeg]

WT

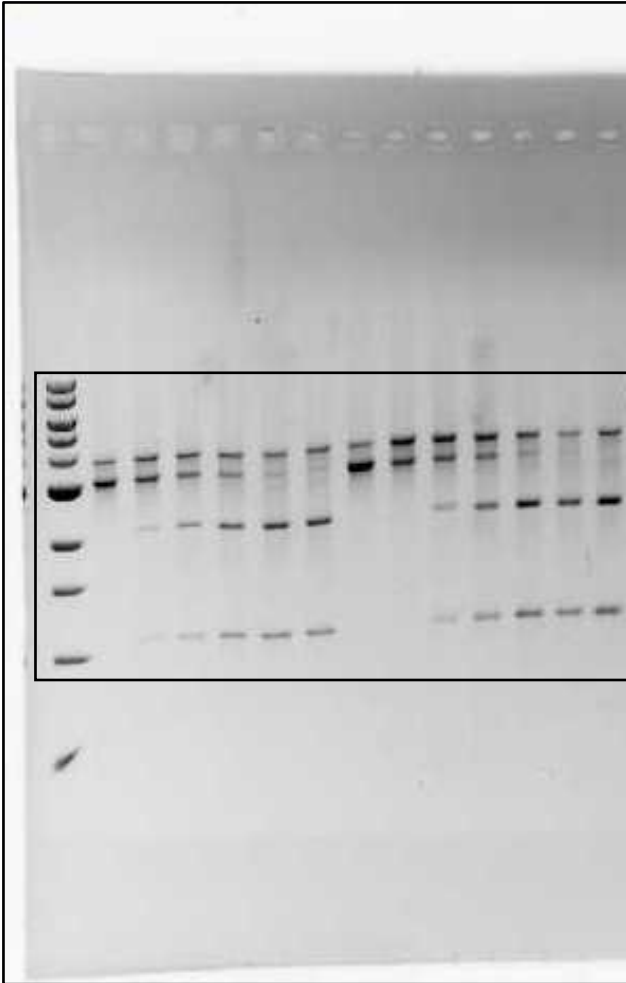

Regions cropped for  
Figure 2B  
See figure for labels

*exo1 $\Delta$  sgs1 $\Delta$*

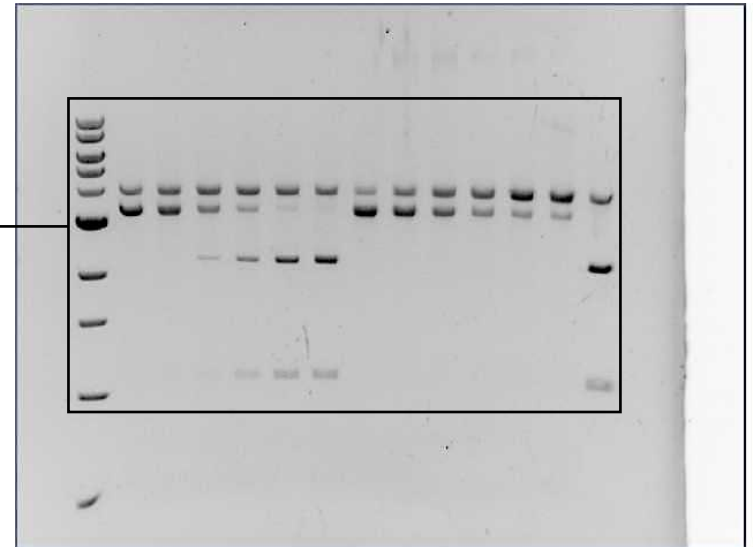

Supplement: Figure 2—source data 1. [file elife-84322-fig2-data1.zip › Figure 2B/Source data- Figure 2B.pdf]

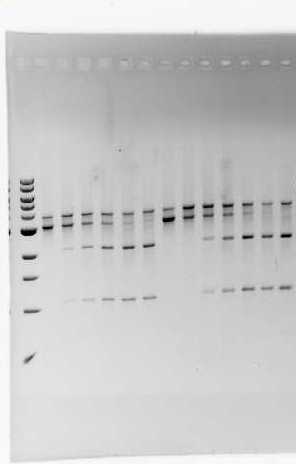

Supplement: Figure 2—source data 1. [file elife-84322-fig2-data1.zip › Figure 2B/Source data- Figure 2B (WT).jpeg]

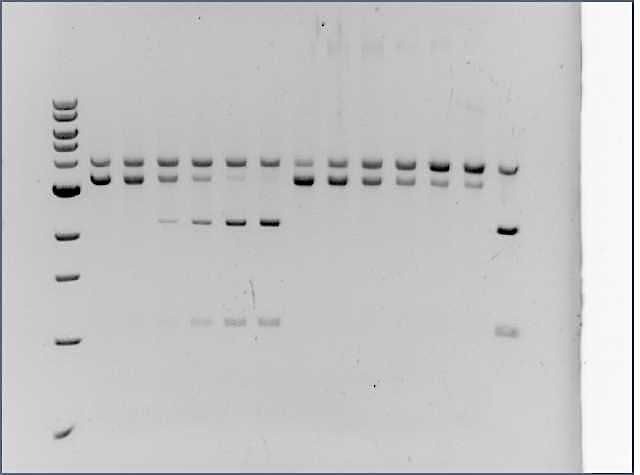

Supplement: Figure 2—source data 1. [file elife-84322-fig2-data1.zip › Figure 2B/Source data- Figure 2B (exo1 sgs1).jpeg]

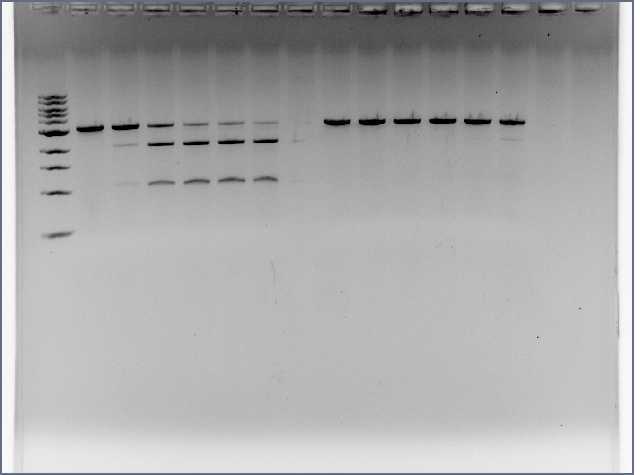

Supplement: Figure 4—source data 1. [file elife-84322-fig4-data1.zip › Figure 4B/Source data- Figure 4B (- Checkpoint).jpeg]

- Checkpoint

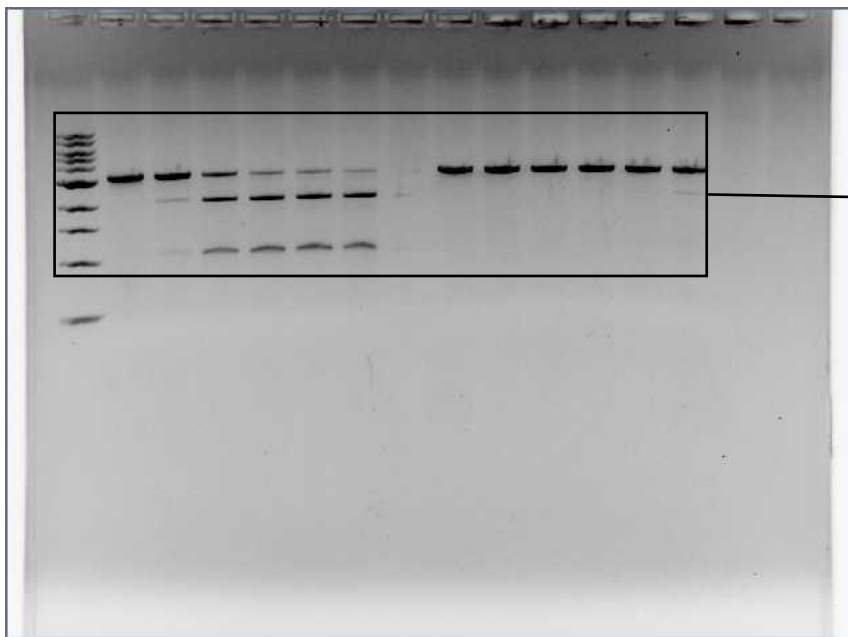

Regions cropped for  
Figure 4B  
See figure for labels

+ Checkpoint

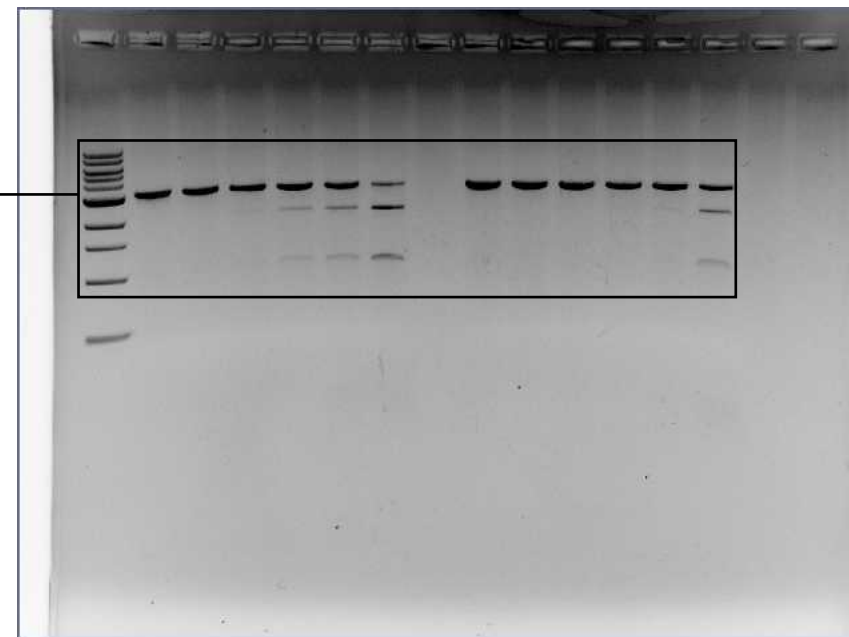

Supplement: Figure 4—source data 1. [file elife-84322-fig4-data1.zip › Figure 4B/Source data- Figure 4B.pdf]

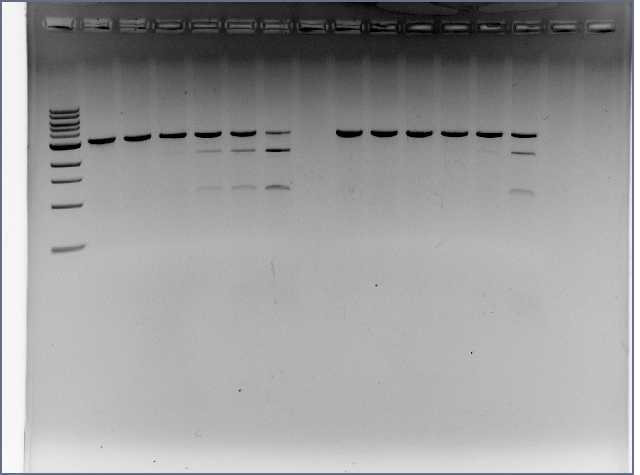

Supplement: Figure 4—source data 1. [file elife-84322-fig4-data1.zip › Figure 4B/Source data- Figure 4B (+ Checkpoint.jpeg]

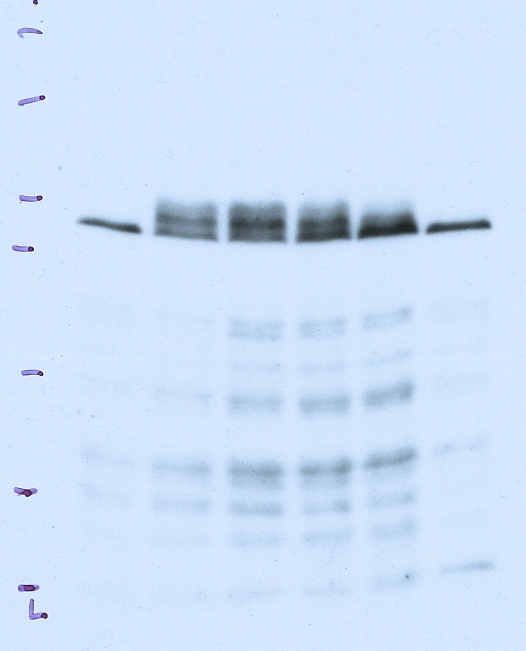

Supplement: Figure 4—source data 1. [file elife-84322-fig4-data1.zip › Figure 4C/Figure 4C- source data (Inter WT blot).jpeg]

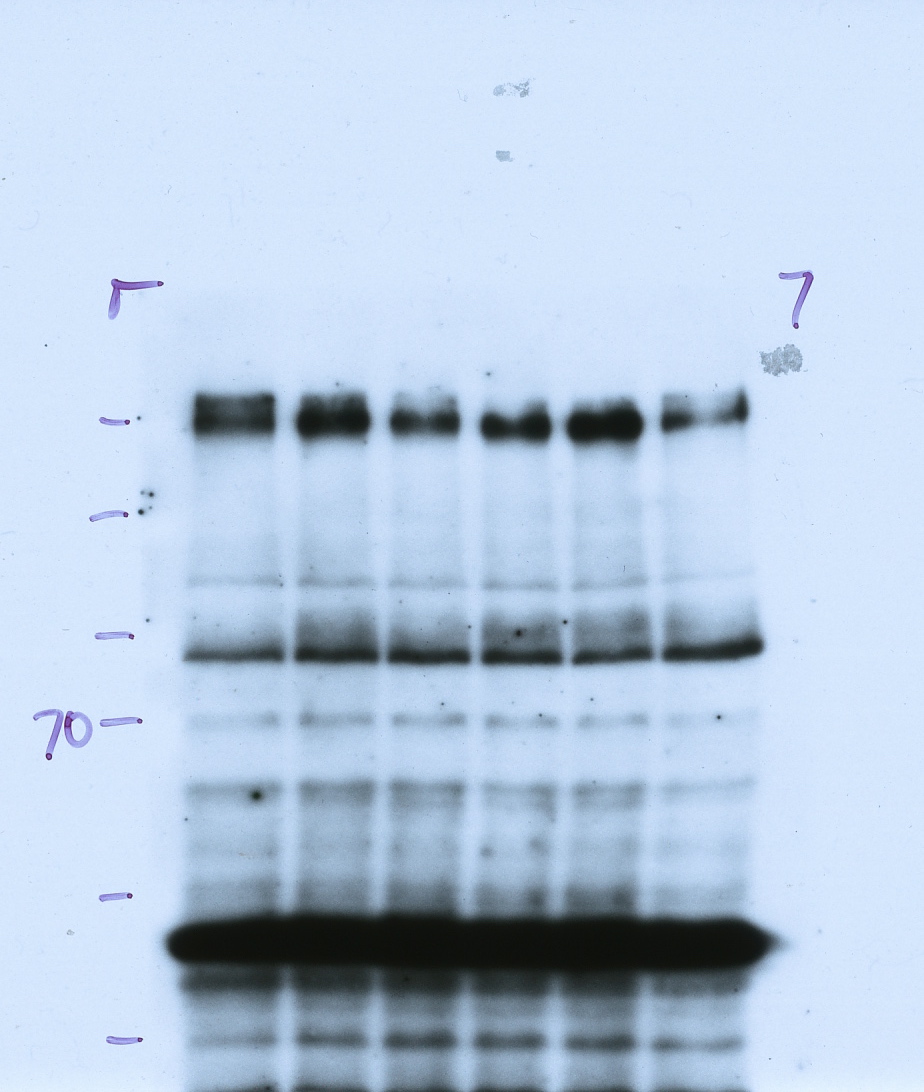

Supplement: Figure 4—source data 1. [file elife-84322-fig4-data1.zip › Figure 4C/Figure 4C- source data (Inter exo1 sgs1 + Chkpt blot).jpeg]

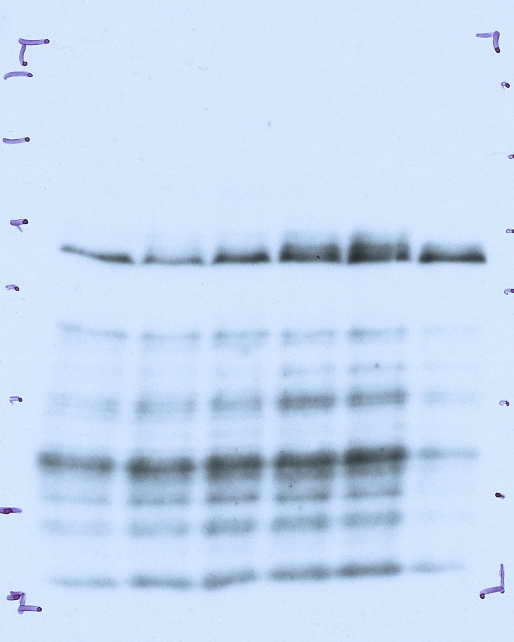

Supplement: Figure 4—source data 1. [file elife-84322-fig4-data1.zip › Figure 4C/Figure 4C- source data (Inter WT + Chkpt blot).jpeg]

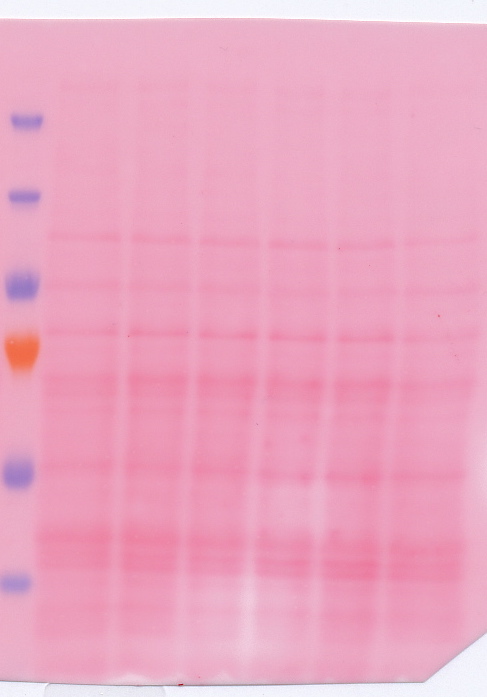

Supplement: Figure 4—source data 1. [file elife-84322-fig4-data1.zip › Figure 4C/Figure 4C- source data (Inter exo1 sgs1 + Chkpt Ponceau stain).jpeg]

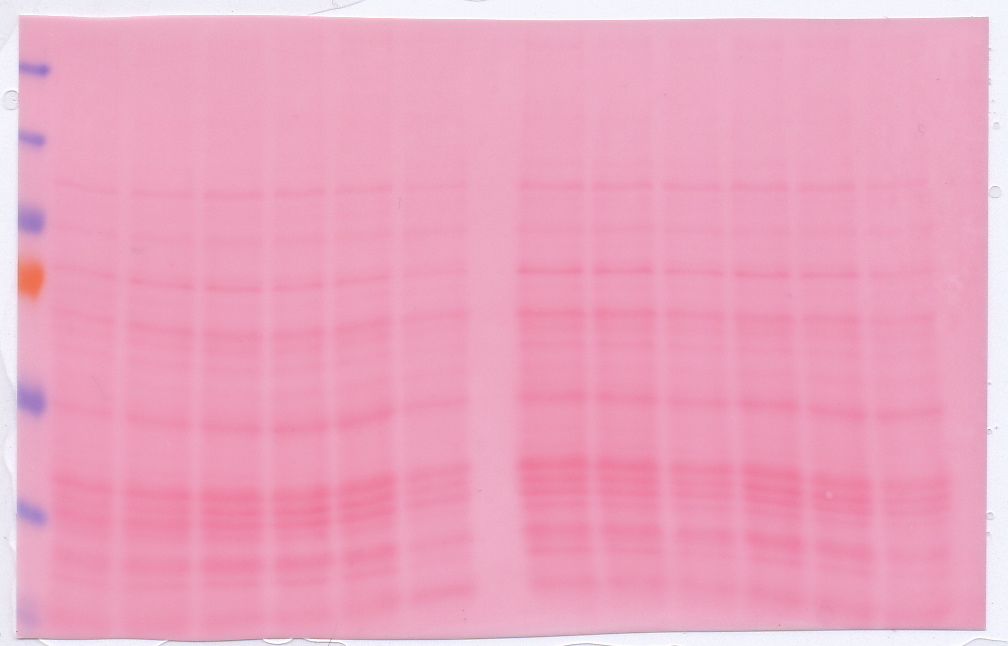

Supplement: Figure 4—source data 1. [file elife-84322-fig4-data1.zip › Figure 4C/Figure 4C- source data (Inter WT [left], exo1 sgs1 [right] Ponceau stain) .jpeg]

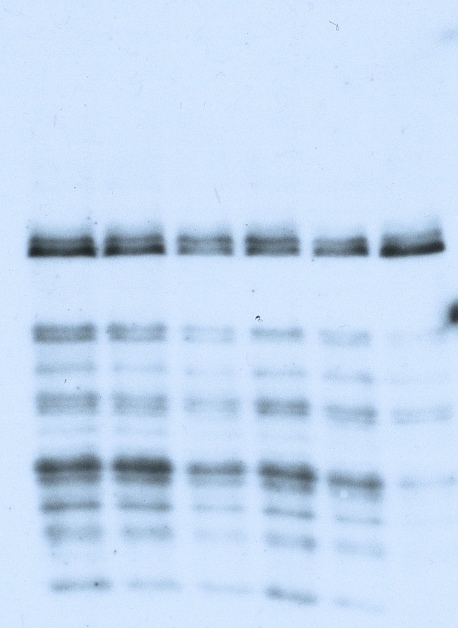

Supplement: Figure 4—source data 1. [file elife-84322-fig4-data1.zip › Figure 4C/Figure 4C- source data (Inter exo1 sgs1 blot).jpeg]

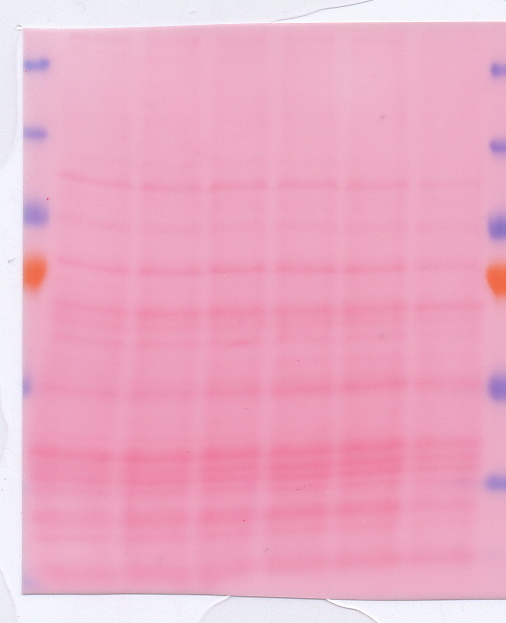

Supplement: Figure 4—source data 1. [file elife-84322-fig4-data1.zip › Figure 4C/Figure 4C- source data (Inter WT + Chkpt Ponceau stain).jpeg]

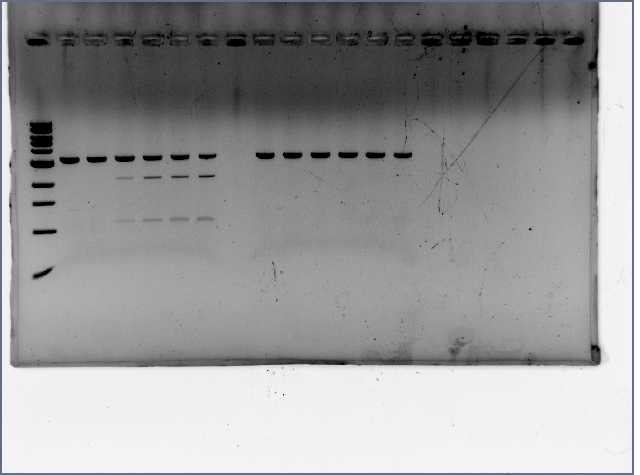

Supplement: Figure 4—source data 1. [file elife-84322-fig4-data1.zip › Figure 4D/Source data- Figure 4D.jpeg]

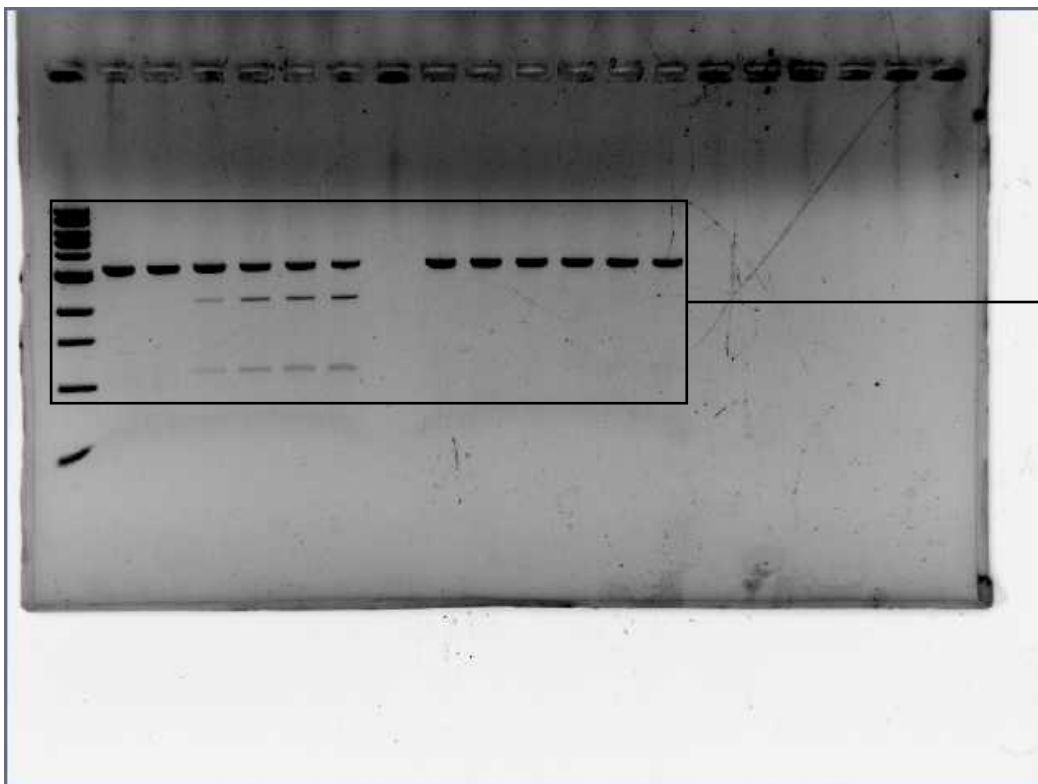

Region cropped for  
Figure 4D  
See figure for labels

Supplement: Figure 4—source data 1. [file elife-84322-fig4-data1.zip › Figure 4D/Source data- Figure 4D.pdf]

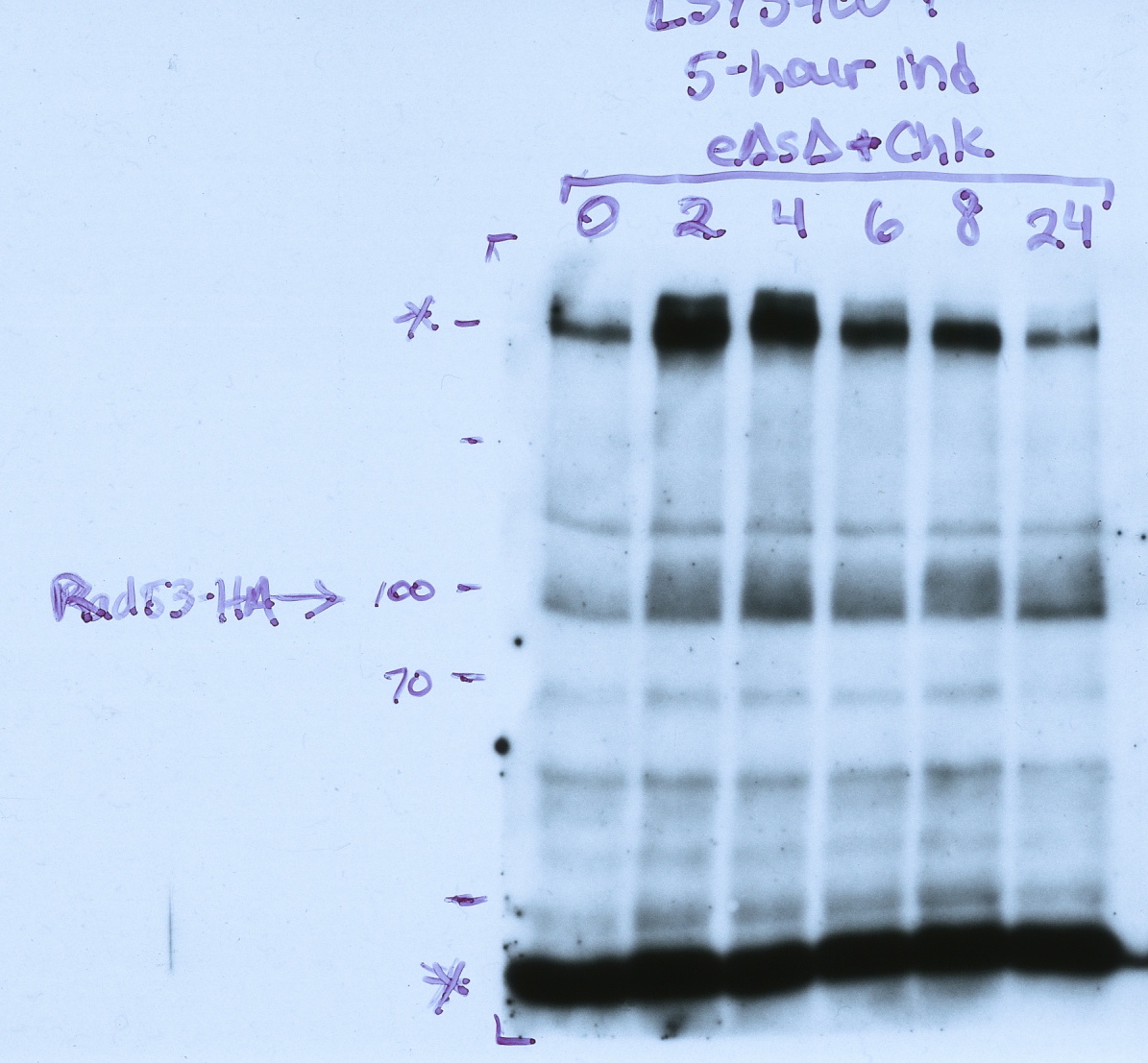

Supplement: Figure 4—source data 1. [file elife-84322-fig4-data1.zip › Fig4_S1D- source data/Fig4_S1D- source data (Inter exo1 sgs1 + chkpt blot).jpg]

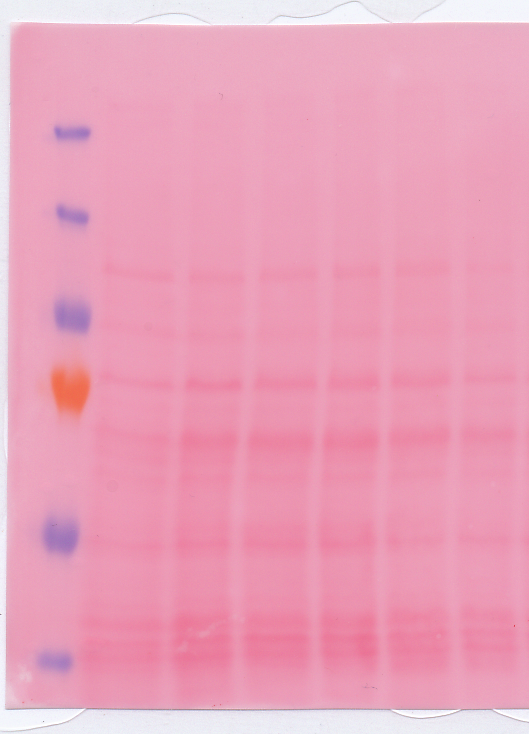

Supplement: Figure 4—source data 1. [file elife-84322-fig4-data1.zip › Fig4_S1D- source data/Fig4_S1D- source data (Inter WT + Chkpt Ponceau stain).tiff]

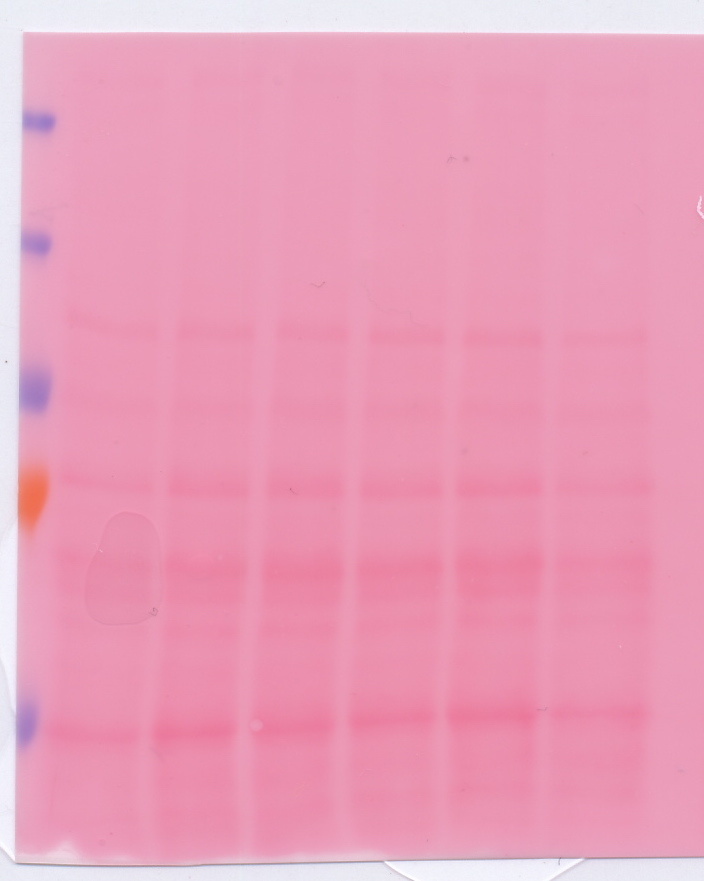

Supplement: Figure 4—source data 1. [file elife-84322-fig4-data1.zip › Fig4_S1D- source data/Fig4_S1D- source data (Inter exo1 sgs1 + chkpt Ponceau stain) .jpg]

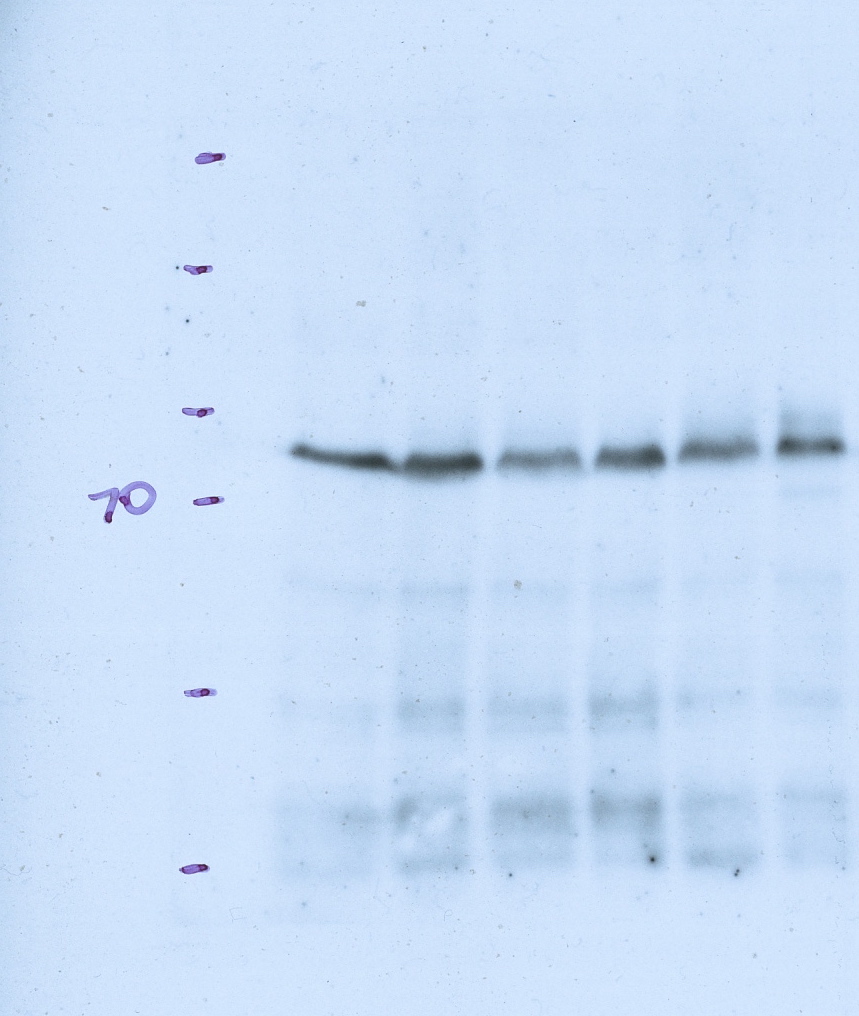

Supplement: Figure 4—source data 1. [file elife-84322-fig4-data1.zip › Fig4_S1D- source data/Fig4_S1D- source data (Inter WT + Chkpt blot).jpeg]

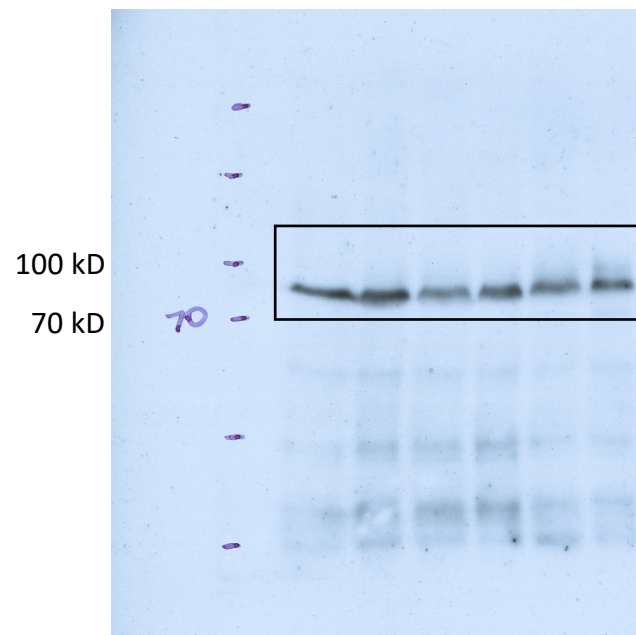

Regions cropped  
for Fig4\_S1D

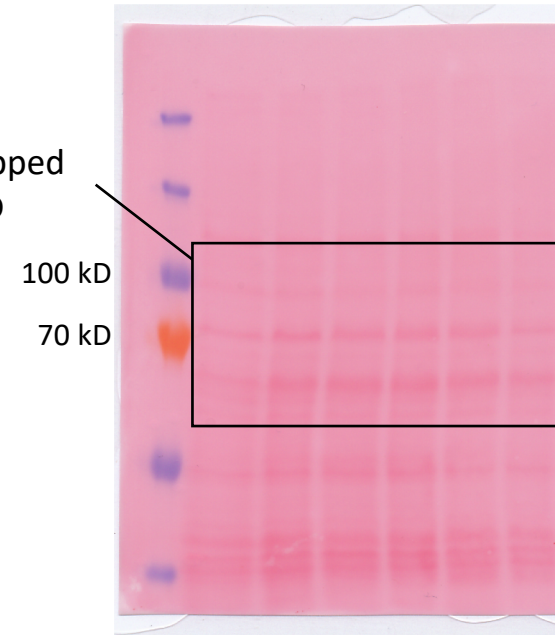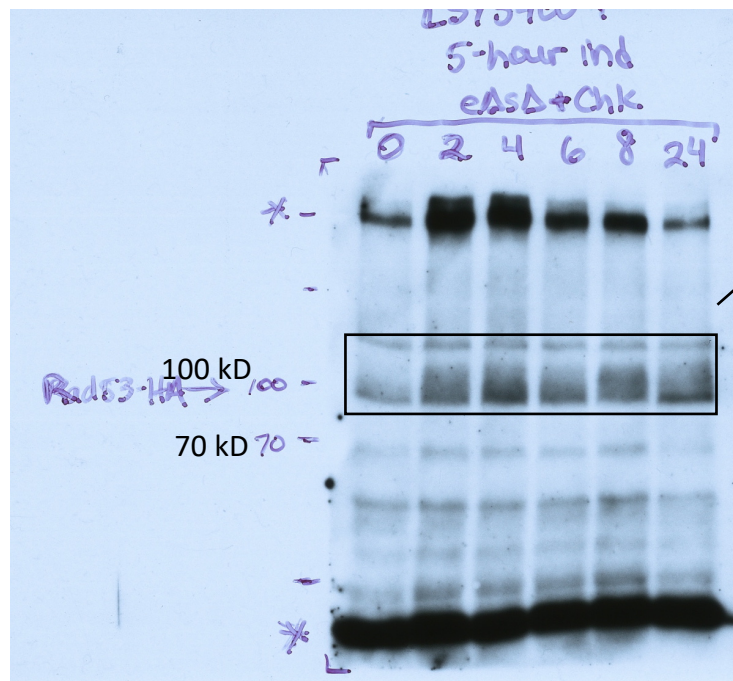

Regions cropped  
for Fig4\_S1D

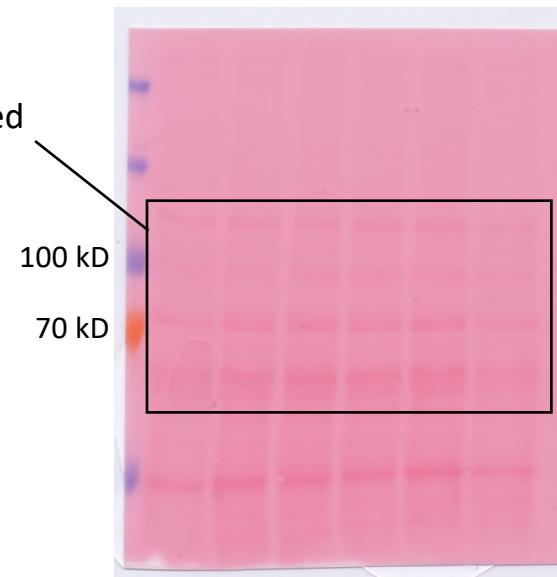

Supplement: Figure 4—source data 1. [file elife-84322-fig4-data1.zip › Fig4_S1D- source data/Source data- Fig4_S1D.pdf]

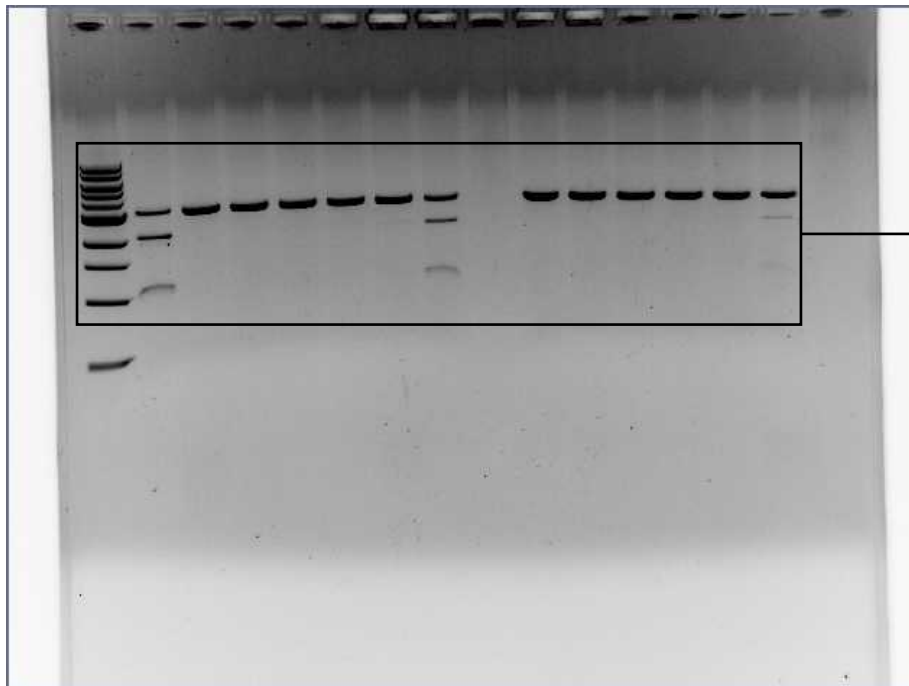

Region cropped for  
Supplementary Fig4\_S1C  
See figure for labels

Supplement: Figure 4—source data 1. [file elife-84322-fig4-data1.zip › Fig4_S1C- source data/Fig4_S1C- source data.pdf]

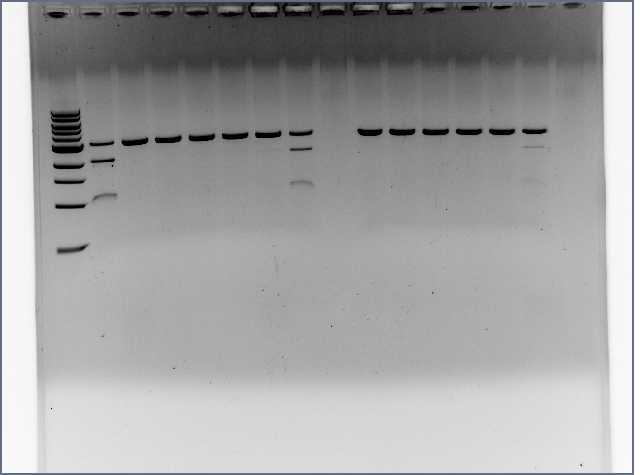

Supplement: Figure 4—source data 1. [file elife-84322-fig4-data1.zip › Fig4_S1C- source data/Fig4_S1C- source data.jpeg]

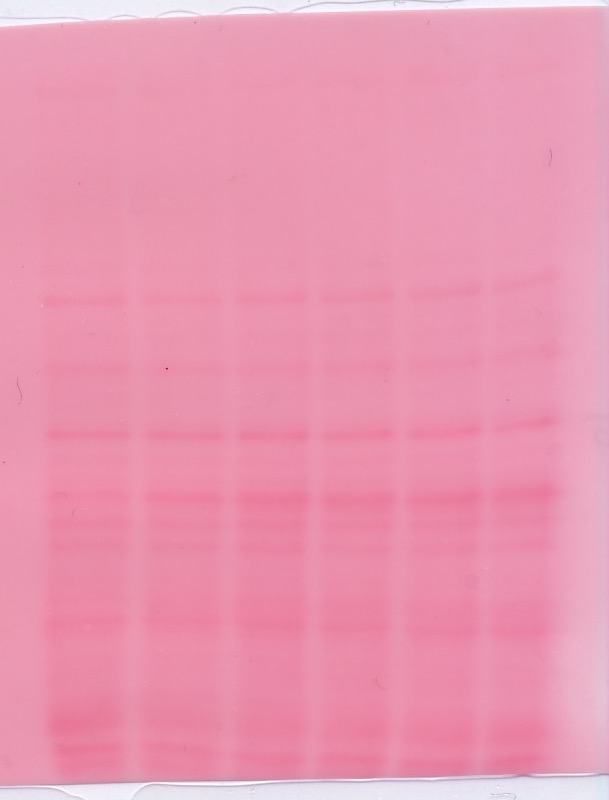

Supplement: Figure 5—source data 1. [file elife-84322-fig5-data1.zip › Figure 5C/Figure 5C- source data (19kb Ponceau stain).jpeg]

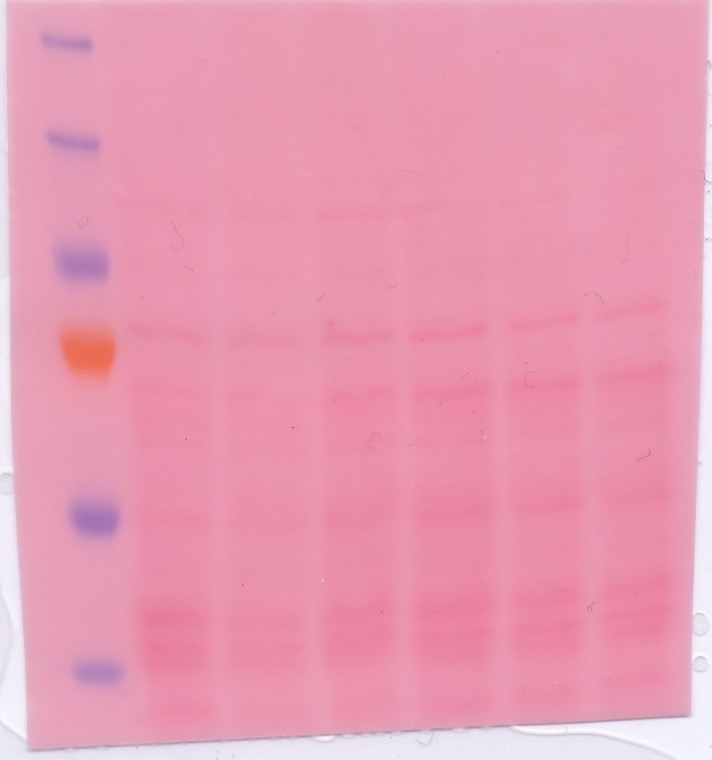

Supplement: Figure 5—source data 1. [file elife-84322-fig5-data1.zip › Figure 5C/Figure 5C- source data (54kb Ponceau stain).jpeg]

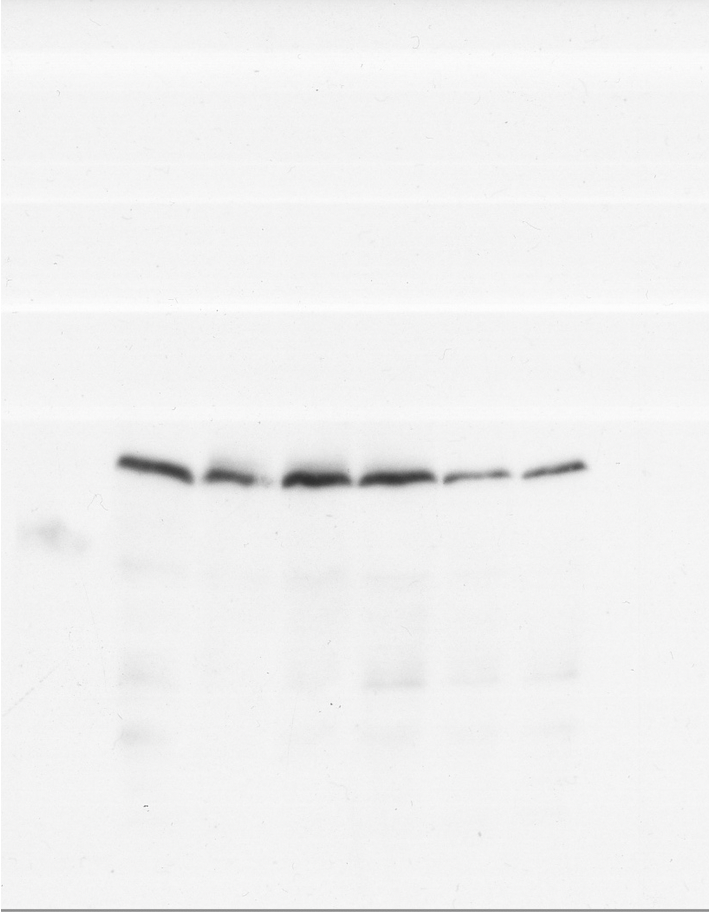

Supplement: Figure 5—source data 1. [file elife-84322-fig5-data1.zip › Figure 5C/Figure 5C- source data (54kb blot).png]

19 kb donor

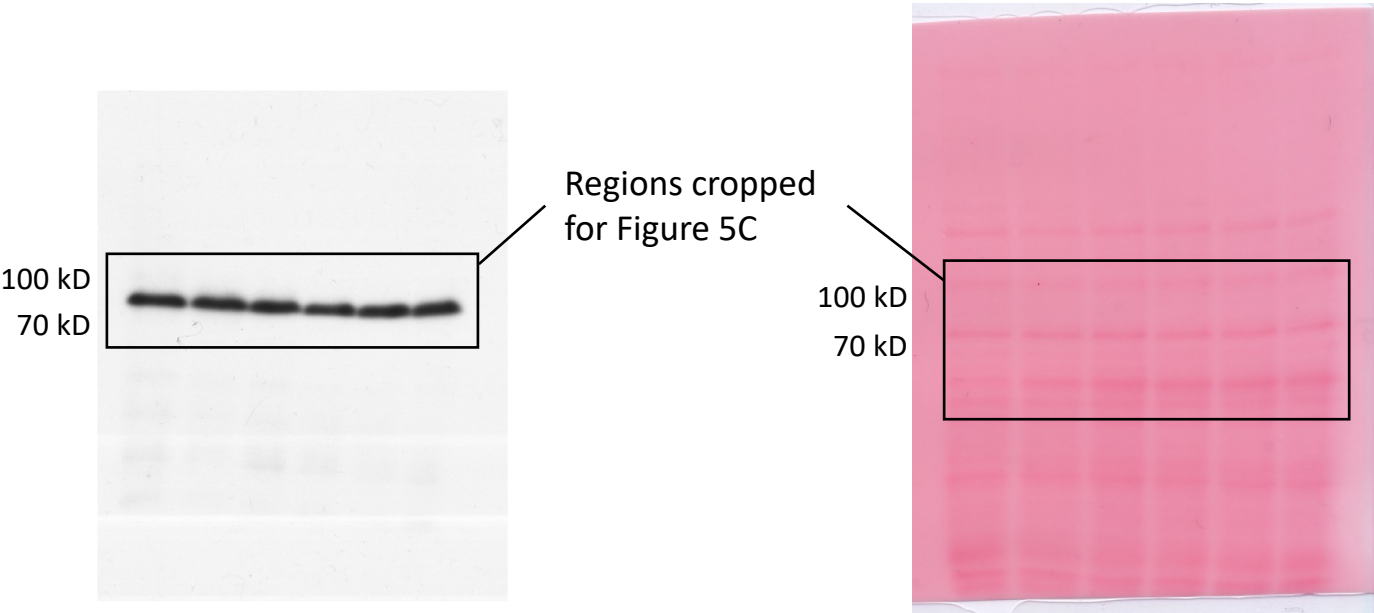

54 kb donor

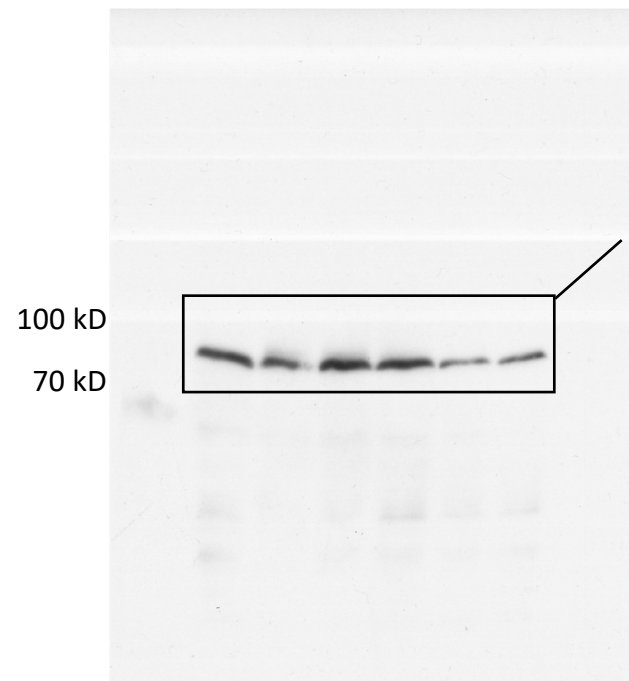

Regions cropped  
for Figure 5C

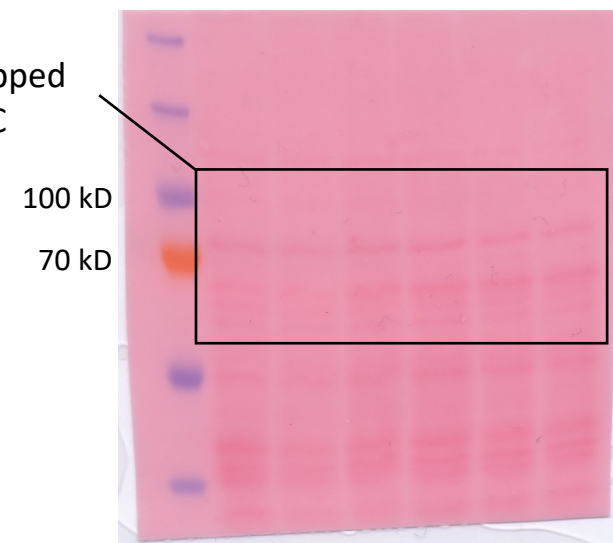

448 kb donor

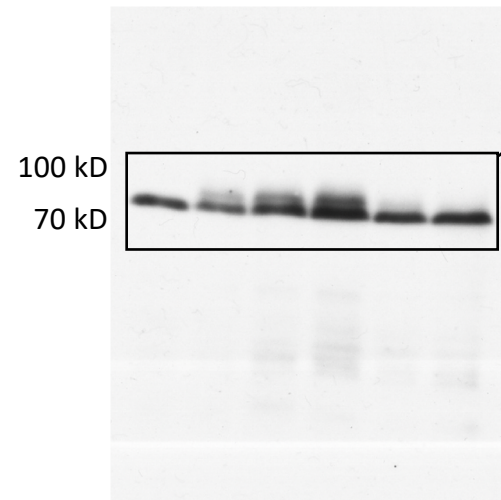

Regions cropped  
for Figure 5C

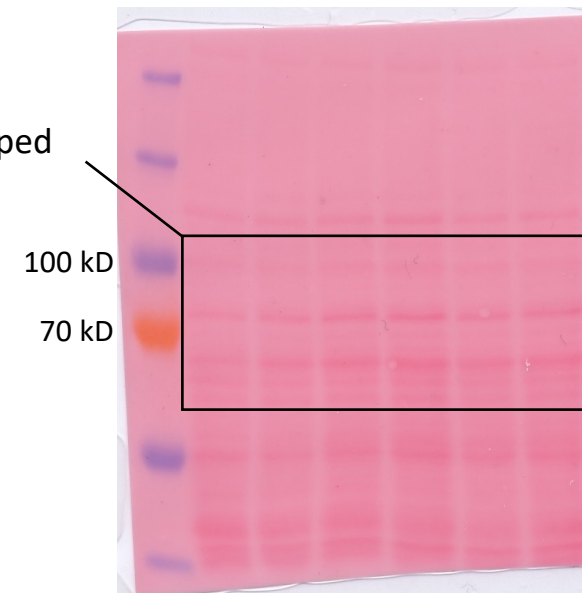

Supplement: Figure 5—source data 1. [file elife-84322-fig5-data1.zip › Figure 5C/Figure 5C- source data.pdf]

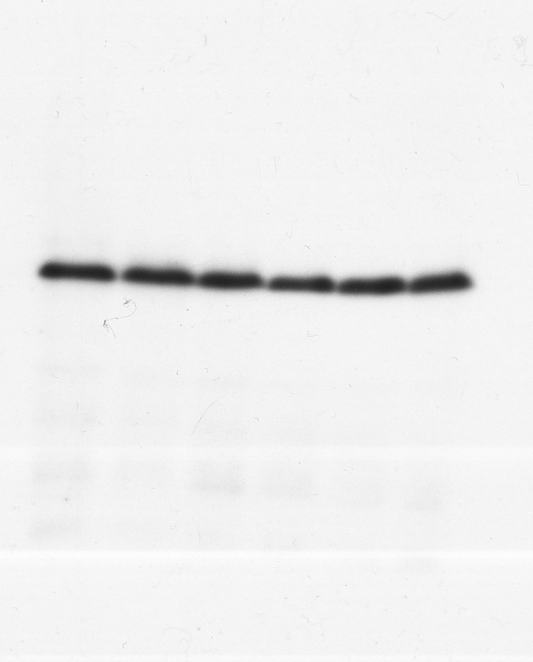

Supplement: Figure 5—source data 1. [file elife-84322-fig5-data1.zip › Figure 5C/Figure 5C- source data (19kb blot).png]

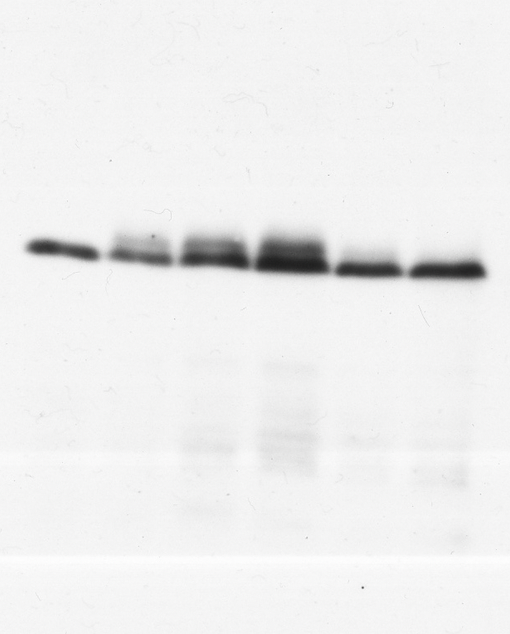

Supplement: Figure 5—source data 1. [file elife-84322-fig5-data1.zip › Figure 5C/Figure 5C- source data (448kb blot).png]

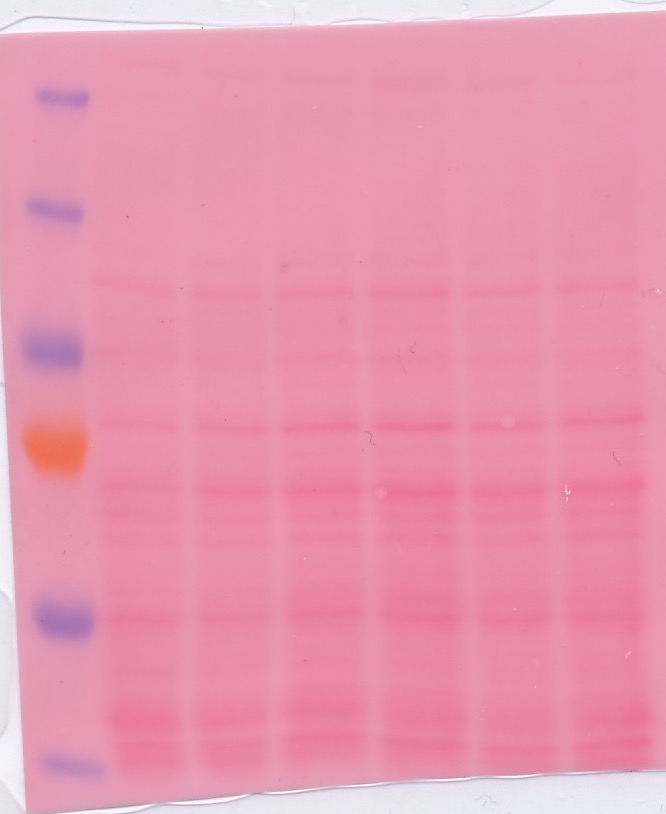

Supplement: Figure 5—source data 1. [file elife-84322-fig5-data1.zip › Figure 5C/Figure 5C- source data (448kb Ponceau stain).jpeg]
