# Supplementary material for: Long-range DNA end resection supports homologous recombination by checkpoint activation rather than extensive homology generation
Source: eLife. 2023 Jun 30;12:e84322. doi: 10.7554/eLife.84322 (PMC10400078; doi:10.7554/eLife.84322)

- checkpoint co-loc

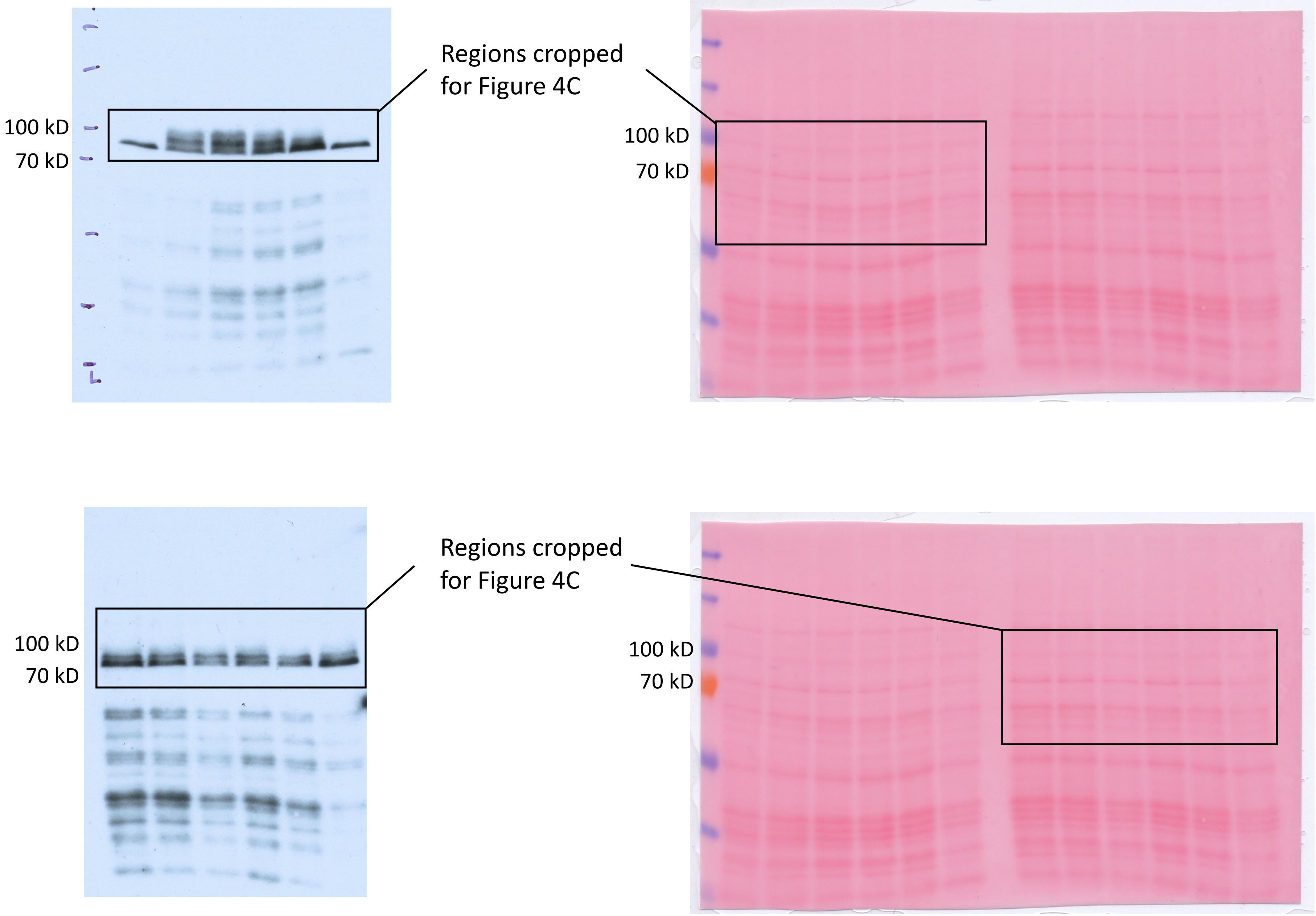

+ checkpoint co-loc

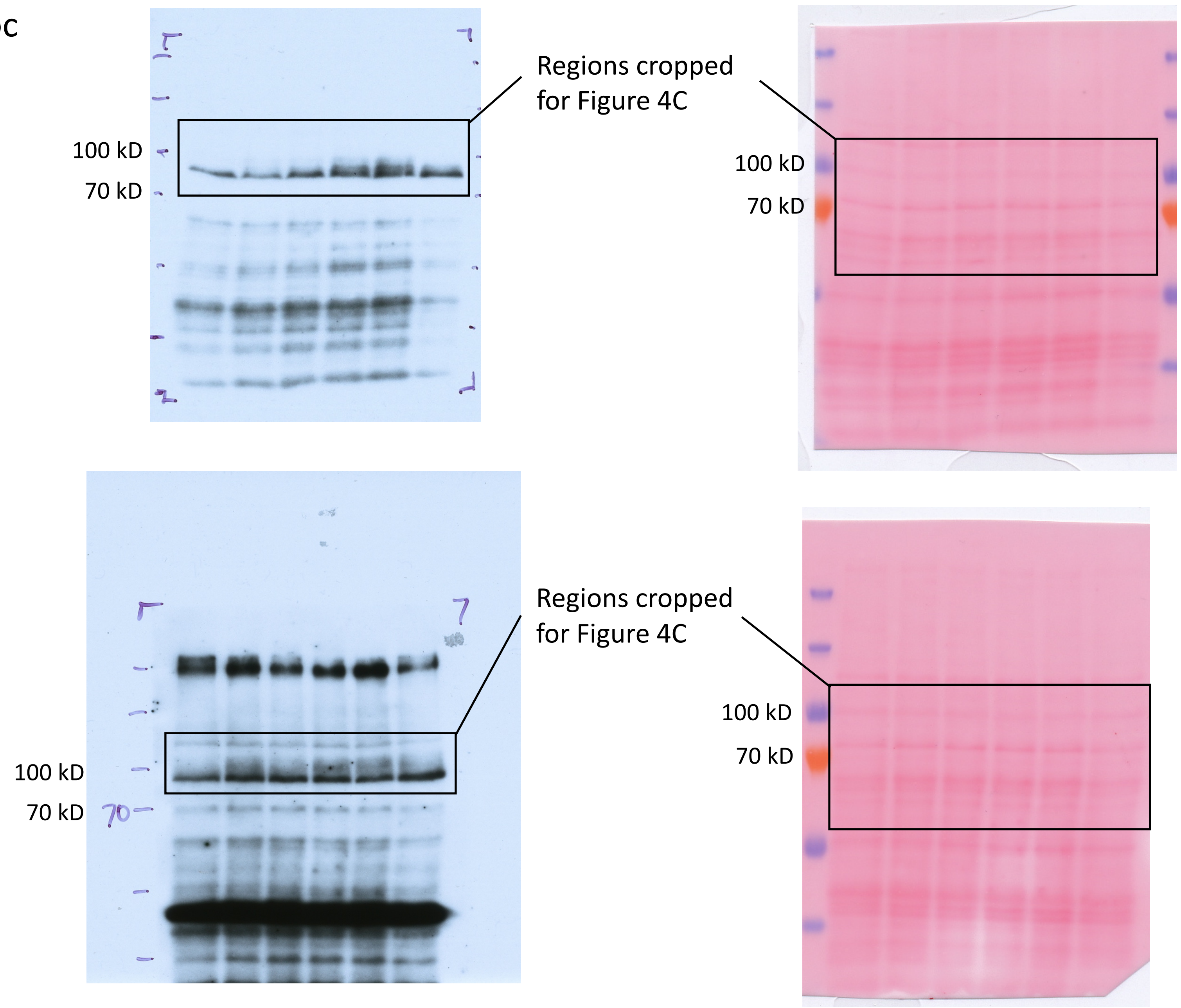

Supplement: Figure 4—source data 1. [file elife-84322-fig4-data1.zip › Figure 4C/Figure 4C- source data.pdf]
